# Induction and transport of Wnt 5a during macrophage-induced malignant invasion is mediated by two types of extracellular vesicles

**DOI:** 10.18632/oncotarget.1336

**Published:** 2013-10-21

**Authors:** Kerstin Menck, Florian Klemm, Julia Christina Gross, Tobias Pukrop, Dirk Wenzel, Claudia Binder

**Affiliations:** ^1^ Dept of Hematology/Oncology, University Medicine, G&ouml;ttingen, Germany; ^2^ Div. Signaling and Functional Genomics, German Cancer Research Center, Heidelberg, Germany; ^3^ Max Planck Institute for Biophysical Chemistry, G&ouml;ttingen, Germany

**Keywords:** Breast cancer, exosomes, Evi, microvesicles, macrophages, Wnt 5a

## Abstract

Recently, we have shown that macrophage (MΦ)-induced invasion of breast cancer cells requires upregulation of Wnt 5a in MΦ leading to activation of β-Catenin-independent Wnt signaling in the tumor cells. However, it remained unclear, how malignant cells induce Wnt 5a in MΦ and how it is transferred back to the cancer cells. Here we identify two types of extracellular particles as essential for this intercellular interaction in both directions. Plasma membrane-derived microvesicles (MV) as well as exosomes from breast cancer cells, although biologically distinct populations, both induce Wnt 5a in MΦ. In contrast, the particle-free supernatant and vesicles from benign cells, such as platelets, have no such effect. Induction is antagonized by the Wnt inhibitor Dickkopf-1. Subsequently, Wnt 5a is shuttled via responding MΦ-MV and exosomes to the tumor cells enhancing their invasion. Wnt 5a export on both vesicle fractions depends at least partially on the cargo protein Evenness interrupted (Evi). Its knockdown leads to Wnt 5a depletion of both particle populations and reduced vesicle-mediated invasion. In conclusion, MV and exosomes are critical for MΦ-induced invasion of cancer cells since they are responsible for upregulation of MΦ-Wnt 5a as well as for its delivery to the recipient cells via a reciprocal loop. Although of different biogenesis, both populations share common features regarding function and Evi-dependent secretion of non-canonical Wnts.

## INTRODUCTION

Infiltrating macrophages (MΦ) are involved in the various steps of tumor progression from primary cancer cell invasion to metastatic colonization of distant tissues [1]. They are characterized by a tumor-promoting M2-profile of secreted cytokines which differs from that of classically activated M1-MΦ by high abundance of IL-10 and −4 and low amounts of IL-1β [2]. TNFα is also critical for the proinvasive effect of MΦ [3] governing the induction of matrix metalloproteinases (MMP) which are necessary for extracellular matrix break-down during invasion [4].

How cancer cells communicate with infiltrating MΦ to elicit a tumor-promoting phenotype is still not completely clear. Several soluble factors have been identified, such as CSF-1, which engages in a reciprocal proinvasive loop with MΦ-derived EGF [5]. We have shown that MΦ-induced invasion of breast cancer cells requires upregulation of Wnt 5a in MΦ and activation of β-Catenin-independent Wnt signaling in the tumor cells [6]. Wnt ligands, especially when signaling through the canonical Wnt/β-Catenin pathway, play a critical role in proliferation and migration during embryonic development [7] as well as in malignant transformation and progression. The β-Catenin-independent ligand Wnt 5a can act either as a tumor promoter [8] or suppressor in a context-dependent way [9]. It is expressed not only in tumor cells, where it regulates MMP transcription and migration, but also during inflammatory reactions of MΦ [10]. The secretion of Wnts requires lipid modification by the acyltransferase porcupine in the endoplasmatic reticulum from where they are shuttled to the Golgi apparatus and transported to the plasma membrane by binding to the seven pass-transmembrane protein Eveness interrupted (Evi) [11]. Distribution of the hydrophobic Wnts outside the cell is still a matter of debate and can occur, among other ways, by attachment to extracellular membranous vesicles [12].

Mammalian cells produce these particles both constitutively as well as following various triggers [13]. Composition, size and function differ according to their biogenesis. Three main populations can be distinguished: exosomes ((Exo) approx. 50 – 100 nm), microvesicles ((MV) 100 – 1000 nm) and apoptotic bodies (1 – 5 μm) [14]. The latter are products of dying cells and contain intracellular organelles or fragments of these. Exo are derived from endosomal vesicles and released via formation of multivesicular bodies (MVB) and fusion with the plasma membrane. In contrast, MV directly bud off from the outer plasma membrane. MV often expose phosphatidyl serine (PS) and tissue factor which explains their procoagulatory function [15], as well as various surface molecules of the respective parent cell [16]. They also contain hydrophilic proteins, frequently enriched in comparison to the parent cell, such as IL-1β, for which they provide a mode of atypic secretion [17]. A similar function has been reported for Exo which export biologically active *Drosophila* Wingless as well as mammalian Wnt 3a in an Evi-dependent way [18]. In neurons, the Evi-Wnt complex can be transferred via exosomal vesicles across synapses, indicating a trafficking function of Evi not only in the Wnt-secreting but also in the accepting cells [19]. An association of Wnt-Evi with MV, particularly in the human system, has not yet been described.

While there is increasing evidence that Exo play a role in Wnt secretion and also in cancer development [20], much less is known on MV in this context. Tumor cell-derived MV have been found in various body fluids of cancer patients [21], [22], their amount correlating with advanced stage. Although their effect has originally been attributed to their prothrombotic activity, recent data indicate a direct involvement in cancer progression. MV induce transformation via horizontal transfer of the mutated EGF receptor [23] and interfere with immune reactions by transmission of MHC molecules [24] and interaction with PS receptors [25]. They modulate the activity of MΦ via transport of HSP 70 [26], induction of IL-10 as part of an anti-inflammatory phenotype [27] and by shuttling of IL-8 and VEGF mRNA [28]. In a murine model of renal carcinoma, MV trigger angiogenesis and promote metastasis [29]. Malignant invasion is enhanced via reciprocal exchange of MV between prostate cancer cells and fibroblasts [30] as well as by MV-induced MMP upregulation [31].

Based on these findings we hypothesized that extracellular particles are attractive candidates for the intercellular communication between cancer cells and infiltrating MΦ. We therefore investigated the role of MV and exosomes from either cell type in MΦ-induced invasion of breast cancer cells and in the regulation and transport of MΦ-Wnt 5a which is crucial for this process.

## RESULTS

### Microvesicles and exosomes are distinct vesicle populations

**Figure 1:**
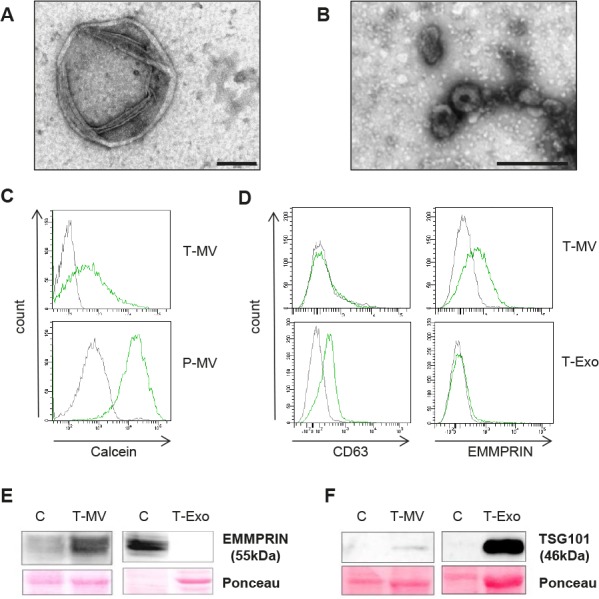
MV and Exo are distinct vesicle populations (A) TEM of MV released from unstimulated SK-BR3 cells (T-MV) showing spherical membrane vesicles with a diameter of >100nm (scale bar = 200 nm). (B) TEM of Exo from the same cells (scale bar = 200 nm). (C + D) Flow cytometry (gray = isotype control, green = stained cells): (C) Calcein staining of T-MV and platelet P-MV confirming the membrane integrity. (D) Analysis of CD63 and EMMPRIN on SK-BR3 vesicles. (E+F) Western blot of EMMPRIN and TSG101 in whole cell lysates (=C) and vesicles (blot exposure time 4-fold longer for C/Exo than for C/MV).

In a coculture system without direct cell-cell contact, we have previously shown that cancer cells induce upregulation of Wnt 5a in MΦ which then leads to enhanced malignant invasion. We now aimed to clarify whether this is mediated by soluble or vesicle-associated components of the supernatant. MV and Exo from breast cancer cell lines (T-MV/-Exo), peripheral blood-derived MΦ (MΦ-MV/-Exo) and donor platelets (P-MV/-Exo) as a control were prepared by differential centrifugation without any previous stimulation. Particular care was taken to obtain non-activated P-MV/-Exo populations by isolating the vesicles directly from the concentrates without any additional stimulation step as used by many other authors. Analysis of the MV fraction by transmission electron microscopy (TEM) yielded membrane-enclosed particles with the typical irregular shape of MV and a diameter between 100 and 1000 nm. The Exo fraction, in contrast, contained smaller, often cup-shaped vesicles (Fig. 1 A, B). There was no evidence of apoptotic bodies, which, according to their size, would appear in the MV fraction. Membrane integrity and the vital nature of the MV were confirmed by staining with calcein AM, fluorescent only after enzymatic metabolization (Fig. 1 C). The tumor vesicles were further characterized regarding the expression of typical proteins (Fig. 1 D – F). CD63 and the cytosolic tumor susceptibility gene TSG101, both considered specific for Exo, were present on T-Exo, but not on T-MV from SK-BR-3 cells. As a marker for T-MV, we identified the plasma membrane-bound extracellular MMP inducer EMMPRIN, which was present exclusively on T-MV. The same results were achieved with MCF-7 vesicles (not shown). Together this indicates that the centrifugation protocol effectively separated the two populations and that they are in fact biologically distinct.

### Tumor cell-derived vesicles induce production of Wnt 5a-positive MΦ vesicles which then induce invasion

**Figure 2:**
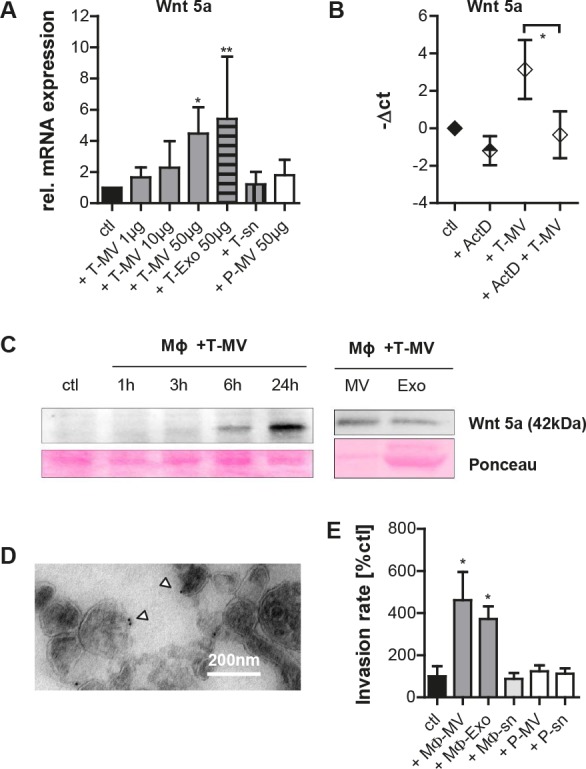
Tumor cell-derived vesicles induce production of Wnt 5a-positive MΦ vesicles which then induce invasion (A) *WNT5A* mRNA is upregulated after 24h in MΦ by T-MV from MCF-7 cells but neither by P-MV nor by the tumor cell supernatant (sn) (qRT-PCR, mean±SD, n=5, *p<0,001, **p<0.01, ***p<0.05). (B) Inhibition of mRNA synthesis in MΦ by ActD (32μM) antagonizes *WNT5A* upregulation by T-MV (qRT-PCR, n=3, p<0.05). (C) Western blot: Upregulation of Wnt 5a in MΦ by 50μg T-MV as well as in MΦ-MV and Exo, isolated after 24 h. (D) Immunogold staining of Wnt 5a on MV from T-MV-stimulated MΦ (TEM). (E) Microinvasion assay. Exposure of MCF-7 cells to 1 μg of particles as indicated induces invasion, while the particle-free supernatant (sn) and P-MV do not (means ± SD, n=3, *p<0,001).

We next asked whether these particles are responsible for the upregulation of Wnt 5a in MΦ which is the critical step during MΦ-induced invasion. Therefore, MΦ were exposed to T-MV and T-Exo from MCF-7 cells. Both vesicles induced Wnt 5a mRNA in MΦ in a concentration-dependent way while the respective supernatant and P-MV as a benign control did not (Fig. 2 A). MCF-7 cells constitutively express very low amounts of Wnt 5a mRNA raising the possibility that the increase in MΦ-Wnt 5a was due to horizontal mRNA transfer. We therefore exposed MΦ to T-MV with concomitant inhibition of mRNA transcription by actinomycin D (ActD) (Fig. 2 B) in concentrations not interfering with MΦ viability. Under these conditions, T-MV did not increase Wnt 5a mRNA, indicating that Wnt 5a in MΦ is newly transcribed. Wnt 5a induction could be confirmed on the protein level. In contrast to unstimulated MΦ, Wnt 5a was increasingly detectable not only in whole MΦ exposed to T-MV but also in the respective MV and Exo (Fig. 2 C). As demonstrated by TEM and immunogold staining, Wnt 5a localized to the outer membrane of these MV (Fig. 2 D). Together, this indicates that Wnt 5a export from MΦ occurs at least partly via extracellular vesicles.

We then analyzed the individual effects on invasion of both kinds of vesicles and the particle-free supernatant derived from MΦ pre-stimulated with T-MV as described above. In Boyden chamber microinvasion assays MCF-7 cells responded with significantly enhanced invasion to addition of these MΦ-MV and Exo (Fig. 2 E), but not to incubation with the respective particle-free supernatant. Equally, P-MV as well as their supernatant had no effect.

**Figure 3:**
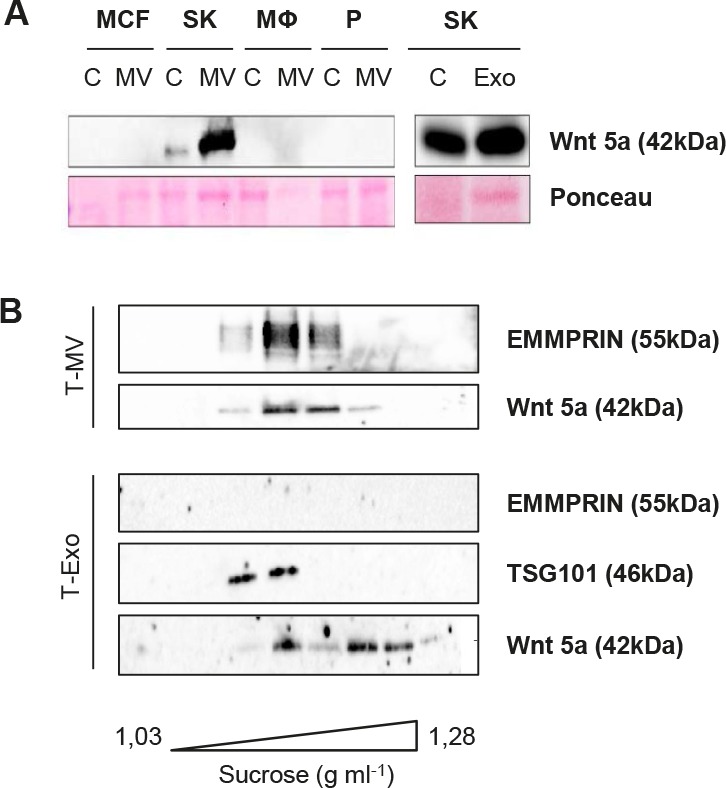
Wnt 5a is also exported on MV and Exo of SK-BR-3 cells (A) Western blot: Wnt 5a is enriched in MV and Exo from SK-BR-3, but absent in MV from MCF-7, unstimulated MΦ and platelets. (B) Sucrose gradient of the T-MV and the T-Exo fraction from SK-BR-3, showing partial colocalization of Wnt 5a with the MV marker EMMPRIN and the exosomal marker TSG 101.

Wnt 5a export on MV and Exo could be identified also in other cell types. Wnt 5a was strongly enriched in MV and Exo from the constitutively Wnt 5a-positive cell line SK-BR-3 (Fig. 3 A). It was absent in MCF-7, platelets, unstimulated MΦ and the respective MV. In a sucrose gradient performed separately for the MV as well as the Exo fraction of SK-BR-3 cells, Wnt 5a colocalized partly with EMMRPIN and partly with TSG 101, underlining that it is present on both vesicle populations (Fig. 3 B).

### Incorporation of tumor vesicles into MΦ is not required for Wnt 5a induction

**Figure 4:**
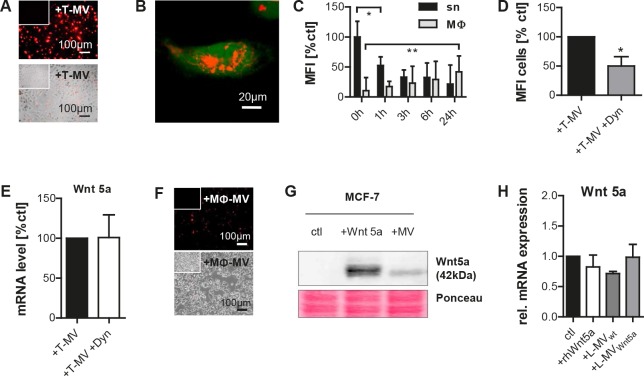
MV are mutually incorporated by MΦ and tumor cells (A-C) Uptake (24h) of PKH26-labeled T-MV from MCF-7 into MΦ: (A) fluorescence/bright field (10×, inserts = controls), (B) confocal microscopy (40×, green = calcein). (C) Quantification of PKH fluorescence in MΦ and sn by flow cytometry (means ± SD, n=5, *p<0,01, **p<0,05). (D) Preincubation (2h) with dynasore (12,5μM) inhibits PKH26-labeled T-MV uptake (flow cytometry, n=3, mean±SD, *p<0,05), but does not counteract T-MV-induced *WNT5A* upregulation in MΦ (E) (qRT-PCR, means ± SD, n=9). (F) Uptake of PKH26-labeled (red) MΦ-MV into MCF-7 cells after 24h (fluorescence/bright field microscopy, 10x, inserts = controls). (G) Western blot: Wnt 5a delivered by MV from Wnt 5a-L cells as well as recombinant human Wnt 5a (100 ng/ml) are detectable in whole MCF-7 lysates. (H) No induction of *WNT5A* mRNA in MCF-7 by rhWnt 5 and MV from wt as well as Wnt 5a-L cells (qRT-PCR, means ± SD, n=9).

We then investigated whether uptake of the tumor vesicles, in particular T-MV, by MΦ is a prerequisite for Wnt 5a induction. After 24 hours, a considerable amount of PKH26-stained T-MV could be identified inside of the MΦ with a respective decrease of fluorescence in the supernatant (Fig. 4 A–C). However, pre-incubation with the endocytosis inhibitor dynasore in concentrations which did not interfere with cell viability (suppl. Fig. 1 A), but effectively inhibited MV incorporation, did not abolish Wnt 5a induction in MΦ (Fig. 4 D, E). This suggests that the contact between vesicles and the MΦ plasma membrane is sufficient to trigger Wnt 5a production. Dynasore alone had no effect on Wnt 5a expression (suppl Fig. 1 B). On the other side of the vesicle-mediated communication, also cancer cells exposed to Wnt 5a-positive MΦ-MV rapidly ingested these particles (Fig. 4 F). To trace the MV-delivered Wnt 5a protein in the receiving cells, we used MV from Wnt 5a-overexpressing L cells (Wnt 5a-L cells). MV-associated Wnt 5a was detectable in recipient MCF-7 cells, as were high doses of recombinant human Wnt 5a used as control (Fig. 4 G). There was no influence of either rhWnt 5a or MV from wild type and Wnt 5a-L cells on Wnt 5a mRNA transcription (Fig. 4 H).

**Figure 5:**
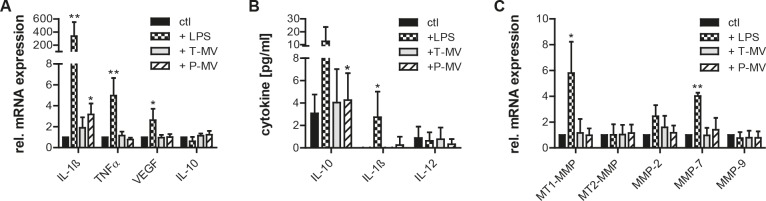
Tumor vesicles do not induce typical M2 features in MΦ (A) qRT-PCR for selected cytokines in MΦ after stimulation with LPS (100ng/ml), T-MV (25μg/ml) and P-MV (25μg/ml) for 24h (mean±SD, n=5, *p<0,05). (B) Cytokine release by MΦ (conditions see above, means±SD, n=5, *p<0,05). (C) mRNA expression of selected MMPs by MΦ stimulated as described above (qRT-PCR, means±SD, n=3, *p<0,05).

To clarify whether T-MV regulate additional secreted factors and induce a shift to a tumor-promoting M2-profile, selected cytokines were measured in MΦ and the supernatant. Expression of the typical M1-markers IL-1ß and TNFα as well as of the M2-markers VEGF and IL-10 did not differ significantly upon exposure to increasing amounts of T-MV. Addition of non-proinvasive P-MV led to a significant increase of IL-1ß mRNA, suggesting a potential shift to an M1-phenotype with anti-tumor functions. However, this could not be confirmed on the protein level in MΦ-supernatants (Fig. 5 A, B). Moreover, there was no effect of T-MV on the mRNA concentrations of various MΦ-derived MMPs (Fig. 5 C).

### Dickkopf-1(DKK-1) antagonizes Wnt 5a induction in MΦ

**Figure 6:**
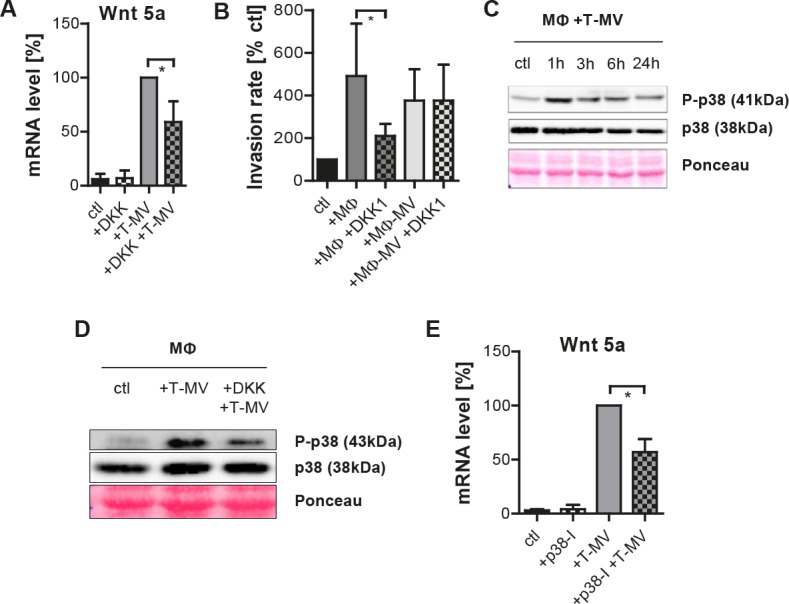
Vesicle-induced Wnt 5a-induction is Wnt- and p38-dependent (A) DKK-1 antagonizes T-MV-mediated (50 μg) *WNT5A* upregulation in MΦ (qRT-PCR, means ± SD, n=5, *p<0,01). (B) DKK-1 (200 ng/ml) inhibits MCF-7 invasion induced by MΦ but not by MΦ-MV (microinvasion assay, means ± SD, n=3, *p<0.001). (C) Phosphorylation of p38 (Western blot) in MΦ + T-MV, which is antagonized by DKK-1 (D). (E) *WNT5A* induction is counteracted by SB-203580 (SB) (qRT-PCR, means ± SD, n=3, *p<0.05).

Earlier, we had identified the Wnt-inhibitor DKK-1 as an antagonist of the complete process of MΦ-induced invasion. To specify its point of action, we separately analyzed its influence on T-MV-mediated Wnt 5a transcription in MΦ as well as on invasion induced by Wnt 5a-positive MΦ-MV. The first step was effectively antagonized by DKK-1 (Fig. 6 A). However, the proinvasive effect of Wnt 5a-positive MΦ-MV was not inhibited by DKK-1, in contrast to invasion induced by coculture with whole MΦ (Fig. 6 B). This suggests that DKK-1 does not so much interfere with the action of Wnt 5a on the recipient tumor cells, but with its production in MΦ. As a downstream event, p38/MAPK was rapidly phosphorylated upon incubation of MΦ with T-MV from MCF-7 cells (Fig. 6 C). This was counteracted by DKK-1 (Fig. 6 D). Underlining the impact of p38 phosphorylation on T-MV-mediated induction of MΦ-Wnt 5a, application of the p38/MAPK inhibitor SB-203580 significantly antagonized this process (Fig. 6 E).

### Wnt 5a associates with vesicles in a non-specific as well as a specific way involving Evi

**Figure 7:**
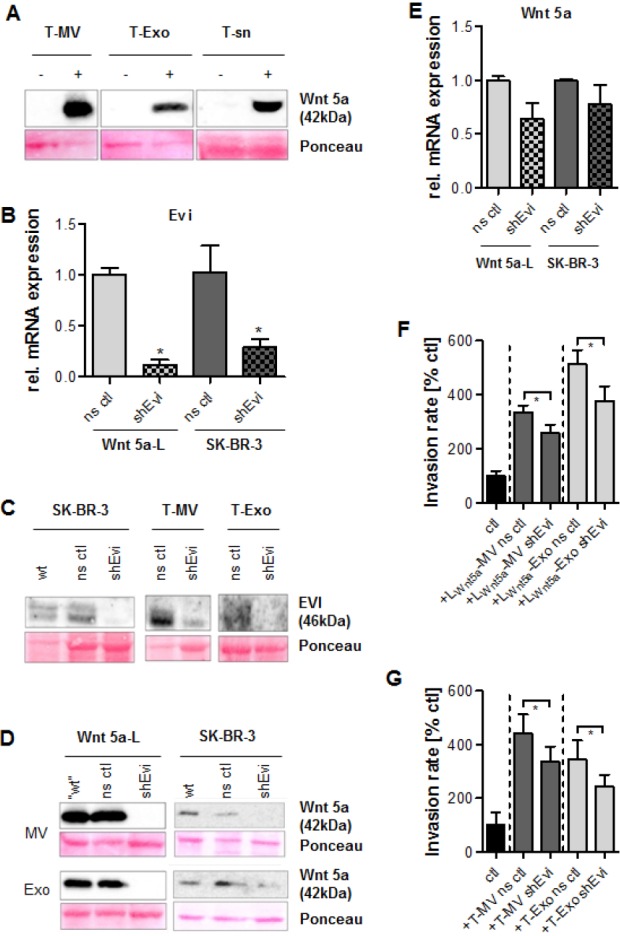
Evi knockdown leads to Wnt 5a depletion on both types of vesicles and inhibits invasion (A) Western Blot: rhWnt 5a (100 ng/ml, 24 h) to some extent associates with MCF-7 vesicles, but also remains in the particle-free supernatant (sn). (B) Stable knockdown of *WLS/EVI* mRNA by shEvi in Wnt 5a-L and SK-BR-3 cells (qRT-PCR, means ± SD, n=3, *p<0.05, ns ctl = nonsense control). (C) Western blot: Downregulation of Evi protein in SK-BR-3 cells and their vesicles by shEvi. (D) Western blot: Wnt 5a depletion in MV and Exo from Evi knockdown cells. (E) Influence of shEvi on *WNT5A* mRNA expression (qRT-PCR, means ± SD, n=3, *p<0.05). (F, G) Microinvasion assays: Evi knockdown antagonizes the increase of MCF-7 invasion induced by MV and Exo from (F) Wnt5a-L and (G) SK-BR-3 cells (means ± SD, n=3, *p< 0.001).

To investigate whether Wnt 5a non-specifically associates with vesicle membranes, Wnt 5a-negative tumor vesicles were exposed to high concentrations of rhWnt5a. After 24 h, a considerable part of the recombinant protein was detectable on MV and Exo, while a significant amount still remained in the supernatant (Fig. 7 A). However, apart from this non-specific attachment, we could also identify a specific component of the vesicle-associated Wnt 5a export. This involves the cargo protein Evi, recently described as essential for the secretion of Wnt 3a on exosomes by a member of our group. We used Wnt 5a-L cells as well as the breast cancer cell line SK-BR-3 as models for Wnt 5a-positive MΦ. Stable downregulation of Evi expression in these cell lines was performed by short hairpin RNA-mediated silencing (shEvi) (Fig. 7 B). Evi knockdown led to almost complete absence of the Evi protein in SK-BR-3 cells and their vesicles (Fig. 7 C). This was accompanied by downregulation of Wnt 5a not only on Exo but also on MV from both cell types (Fig. 7 D). This seems surprising since plasma membrane-derived MV – as we have shown before – are distinct from Exo and differ from these in their biogenesis and their cargo.

The shEvi effect on Wnt 5a was restricted to the protein since there was no significant reduction of Wnt 5a mRNA in both SK-BR-3 and Wnt 5a-L cells (Fig. 7 E). On the functional level, Wnt 5a depletion of MV and Exo from either cell line was followed by a significant decrease of vesicle-induced invasion of MCF-7 cells (Fig. 7 F, G), indicating that vesicle-associated Wnt 5a is essentially involved in MΦ-induced invasion.

**Figure 8:**
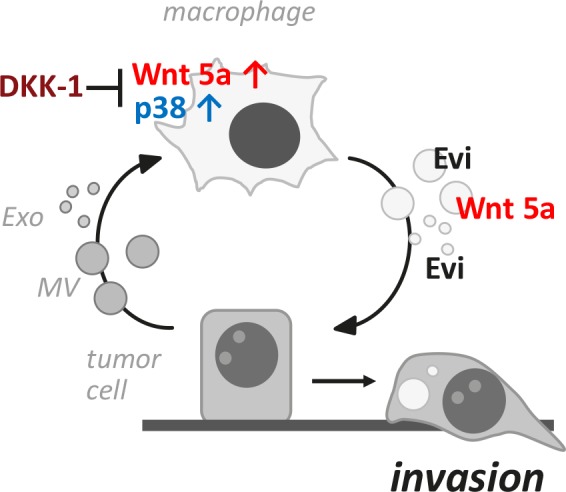
Schematic presentation of the vesicle-mediated reciprocal loop during macrophage-induced tumor cell invasion

### DISCUSSION

Previously, we have shown that MΦ-induced cancer cell invasion depends on Wnt 5a induction in MΦ as an essential part of their proinvasive phenotype. Now we demonstrate that this is not mediated by cancer cell-derived soluble factors but by extracellular vesicles. MV and Exo from benign cells do not induce Wnt 5a in MΦ.

Surprisingly, we identified Wnt 5a, induced by T-MV and T-Exo, not only in whole MΦ but also in MΦ-MV and Exo which are produced in response. Equally, Wnt 5a-L cells and the Wnt 5a-positive breast cancer cell line SK-BR-3 showed enrichment of Wnt 5a in their vesicles. The TEM images of MΦ-MV suggest that Wnt 5a is located at the MV membrane. Wnts are able to non-selectively “stick” to membranes due to their lipid modifications [12]. Consistently, we show that Wnt 5a-negative tumor vesicles can be loaded with recombinant Wnt 5a, part of which remains soluble in the supernatant. Thus, Wnt 5a may indeed non-specifically associate with MΦ vesicles after secretion by stimulated MΦ. However, our experimental evidence suggests that there is also a specific route of Wnt 5a excretion through extracellular vesicles involving the protein Evi. This protein present on Exo and MVB has recently been reported to provide an alternative way of Wnt 3a secretion [18]. Until now, a role of Evi in the secretion of non-canonical Wnts as well as an association with MV has not been described. Here, we show that Evi is expressed not only on Exo but also on MV, a particle population considered biologically different. Knockdown of Evi blocks the export of Wnt 5a via MV in the same manner as via Exo indicating that it is active in both fractions and may be essential for the export of canonical as well as non-canonical Wnts in general.

We took particular care to rule out insufficient purity and mutual contamination of the particle preparations by using an elaborate centrifugation protocol followed by an additional filtration step to separate MV from the smaller Exo. The MV preparations did not contain particles <100 nm, however, an overlap in the range of 100 – 200 nm cannot be completely excluded. Recently, it has become a matter of debate whether Exo and MV biogenesis can be strictly distinguished. Exosomal and endosomal proteins have been found enriched in certain regions of the plasma membrane which can serve as sites of direct Exo generation [32]. However, we identified a different pattern of protein expression on Exo and MV. The plasma membrane-bound EMMPRIN could be established as a specific marker of MV while TSG101, implicated in the biogenesis of MVB, was present predominantly on Exo. Equally, CD63 expression was restricted to Exo. This strongly suggests that we indeed obtained two populations with different biological characteristics and that Evi plays a role in both.

These extracellular particles are critical mediators of MΦ-induced invasion as proven by the fact that the respective particle-free supernatant had no effect, although it should still contain all of the soluble factors we have identified earlier as indispensable for invasion [33]. Obviously, MΦ-derived MV and Exo serve as carriers of Wnt 5a to the tumor cells where, after uptake, it becomes detectable intracellularly. Wnt 5a on these particles is biologically active and essential for induction of tumor cell invasion since Wnt 5a-negative P-MV do not enhance invasion, and Wnt 5a depletion via Evi knockdown antagonizes the proinvasive effect. The fact that invasion induced by Wnt 5a-positive MV from SK-BR-3 cells is only partially reversed by Wnt 5a depletion implicates that there are additional vesicle-associated factors involved.

The way how T-MV and T-Exo, in contrast to vesicles of benign provenience, selectively induce Wnt 5a in MΦ and whether they act at the cell membrane or intracellularly is still not completely clear. MV are known to be non-specifically ingested via endocytosis [34]. Consistently, MΦ rapidly incorporated T-as well as P-MV. However, MV uptake alone did not decide on Wnt 5a induction since this was caused only by T-MV. Moreover, inhibition of endocytosis by dynasore did not counteract Wnt 5a upregulation and subsequent export. This argues against a similar effect as recently described for fibroblast-induced cancer cell motility [35]. There, fibroblast-derived Exo were internalized into breast cancer cells, loaded with cancer cell-derived Wnt 11 and then recycled to activate non-canonical Wnt signaling in these cells. Our findings, in contrast, suggest a still to be defined ligand-receptor interaction at the MΦ surface.

Members of the Wnt family are potential candidates for this interaction since we demonstrate here that the Wnt inhibitor DKK-1 antagonizes vesicle-triggered Wnt 5a induction. Previously, we reported that DKK-1 counteracts tumor cell invasion induced by coculture with whole MΦ. However, it remained unclear how DKK-1, considered predominantly an antagonist of β-Catenin-dependent Wnt signaling, could inhibit MΦ-induced invasion which was mediated by non-canonical Wnt signaling via activation of JNK in the tumor cells [6]. Our present data suggest that DKK-1 exerts its main effect not on the tumor cells but on MΦ where it blocks the shift to a Wnt 5a-positive proinvasive phenotype as well as the production of Wnt 5a-carrying particles. Consistently, it does not antagonize invasion induced by Wnt 5a-positive MΦ-MV from prestimulated MΦ. Our observation that exposure of MΦ to T-MV leads to p38/MAPK phosphorylation which has been implicated in β-Catenin-dependent Wnt signaling [36] may further support this assumption. P38 phosphorylation is effectively counteracted by DKK-1. Inhibition of p38 phosphorylation by a specific antagonist, in turn, downregulates Wnt 5a induction.

In conclusion, extracellular vesicles and not soluble factors as expected are the essential communicators between MΦ and cancer cells during MΦ-induced malignant invasion. Surprisingly, both populations, although of different origin, exert a similar biological function. As shown in figure 8, they partake in a reciprocal loop where malignant MV and Exo induce production of Wnt 5a in MΦ, a step which is sensitive to Wnt antagonists. MΦ-derived Wnt 5a is then delivered via responding MΦ-MV and Exo to the recipient cells and enhances invasion. Transport of Wnt 5a in both populations may occur partly in a non-specific way but is also dependent on the presence of Evi. Thus, Evi seems involved in the secretion of canonical as well as non-canonical Wnts and exerts its cargo function in a more general fashion on extracellular vesicles with different biological characteristics.

## MATERIAL AND METHODS

### Cells, viability and lentiviral production

The human breast cancer cell lines MCF-7 and SK-BR-3 (ATCC) were grown in RPMI-1640 (PAA) + 10% fetal calf serum (FCS). Murine L cells overexpressing Wnt5a (Wnt 5a-L cells) were maintained in DMEM (Biochrom) + 10% FCS. Human MΦ were derived from peripheral blood mononuclear cells as described [6] and cultured in RPMI-1640 + 1% FCS. Viability upon treatment with ActD (32μM, Lundbeck Pharmaceuticals Ireland Limited), DKK-1 (200 ng/ml, R&D systems) and SB-203580 (0,5 μM, Calbiochem) was tested with the MTT assay using standard protocols. For generation of SK-BR-3 and Wnt5a-L cells with stable shEvi expresssion HEK293T cells were cotransfected with the packaging plasmids pVSG-G and pCMVΔR8.91 and the pLKO shRNA control or shEvi plasmid (Sigma) through calcium phosphate precipitation. The ns ctl sequence is 5′-CCCGTGTAAATATGTACATTT-3′, the Evi targeting sequence is 5′-GATCTACAAGTTGACCCGCAA-3′. The virus-containing supernatant was concentrated using lentiviral enrichment reagent (MobiTech). Cells were selected in medium with 2 μg/mL (SK-BR-3) or 15 μg/ml (L cells) puromycin (Sigma).

### Isolation of MV and Exo, and calcein staining

Cells were cultured up to 48h in medium + heat-inactivated FCS (particle-free through ultracentrifugation, 100.000 g, overnight). Supernatants were centrifuged at 750 g (5 min) and 1,500 g (15 min) to remove cells and debris, followed by ultracentrifugation (14,000 g, 35 min, 4°C) to precipitate MV. Pellets were resuspended in 200μl PBS or RIPA lysis buffer for protein quantification (D_c_ protein assay, Bio-Rad). MV membrane integrity was determined by staining with calcein AM (MobiTec). The signal was detected by flow cytometry and related to the acetone-treated negative controls. P-MV were isolated from expired (<2 days) platelet concentrates from healthy blood donors following the same protocol. For Exo preparations, the supernatant after MV precipitation was filtered (0,22 μm, Sarstedt) and ultracentrifuged at 100.000 g (4°C, 2 h). The Exo pellet was washed once in PBS before protein quantification.

### Flow cytometry

Exosomes were coupled to latex beads (aldehyde sulfate, 4 μm, Invitrogen) as described previously [37]. Briefly, exosomes and beads were incubated in MES buffer (0,025 M MES, 0,154 M NaCl, pH 6) for 1h at room temperature before incubating overnight at 4°C with gentle shaking. Beads were blocked with 200 mM glycine for 30 min and washed twice in PBS + 1% FCS (particle-free). MV and exosomes were stained with primary antibodies against EMMPRIN (#sc-13976, Santa Cruz) or CD63 (#556019, BD), washed twice in PBS and incubated with FITC-labeled anti-rabbit (#406403, BioLegends) or anti-mouse (#sc-2010, Santa Cruz) secondary antibodies. Signals were detected with a FACS Canto II flow cytometer. MV and exosome populations were gated and analyzed with the FACS Diva (version 6.1.3.) software.

### Quantitative real-Time RT-PCR

MΦ and MCF-7 cells were stimulated for 24 h with 100 ng/ml LPS (Sigma-Aldrich) or MV/Exo at the indicated concentrations. Total RNA was extracted with the High Pure RNA Isolation kit (Roche) and reverse transcription was performed from 1 μg of total RNA with the iscript cDNA synthesis kit (Bio-Rad). The cDNA products were subjected to quantitative RT-PCR with SYBR green detection (7900 HT system, Applied Biosystems). The primer sequences are listed in suppl. Tab. 1 or for *WNT5A* published in [8].

### Western Blot, ELISA and sucrose gradient

Cells were lysed and homogenized in RIPA lysis buffer (150 mM NaCl/ 0,1% SDS/ 0,5% Na-deoxycholate/ 1% Triton X-100/ 50 mM Tris, pH 7,2). Up to 50 μg of total protein were subjected to SDS-PAGE (8%) and blotted onto a nitrocellulose membrane (Amersham Biosciences). Ponceau S staining was used as loading control. Membranes were incubated with antibodies specific to EMMPRIN (#sc-13976, Santa Cruz), Wnt5a (#MAB645, R&D systems), TSG101 (#7964, Santa Cruz), EVI (#MABS87, Millipore), p38 MAPK total protein (#9212) and phospho-p38 MAPK (#9211), both from Cell Signaling. Signals were detected with ECL™ Prime (Amersham Biosciences). Cytokine concentrations in the supernatants were analyzed with Legend Max™ ELISA kits (BioLegend, IL-1ß #437007, IL-12 p70 #431707, IL-10 #430607) according to the manufacturer's instructions. LPS (100 ng/ml, *E. coli* K-235, Sigma) was used as a positive control. For sucrose gradient preparations, SK-BR-3 supernatant was centrifuged at 750g and 1500g as described above and directly applied to ultracentrifugation (see isolation of MV and Exo). The pellet was washed in PBS and layered on top of a sucrose step gradient (0,25-2,25M) which was ultracentrifuged for 16h at 100.000g. Eight fractions (2ml) were collected and precipitated with ice-cold acetone (1:10, v/v) overnight at −20°C.

### Electron microscopy

MV and Exo pellets were resuspended in 0,1 M sodium phosphate + 2% paraformaldehyde, applied to carbon-coated EM grids (400 mesh, Plano GmbH) and postfixed in 1% glutaraldehyde. The grid was washed with dH^2^O and incubated in 1% uranyl acetate (30 s) for negative contrasting. MV were visualized using the CM 120 Bio Twin transmission electron microscope (Philips) and iTEM software (Olympus). For detection of Wnt5a, MV pellets were stained with above named Wnt5a antibody and an immunogold-labeled secondary antibody (#810.077, Aurion).

### Microinvasion assay and MV uptake

Invasion was quantified using a modified Boyden chamber assay as described [32]. For uptake, tumor cells were exposed to MV/Exo in the indicated concentrations and the according particle-free supernatants. MV uptake was measured using MV stained with the red-fluorescent membrane dye PKH26 (Sigma-Aldrich) according to the manufacturer's instructions. Staining efficiency was tested by flow cytometry. Cells were incubated with the labeled MV (5 μg/ml, 24 h) and analyzed by confocal/fluorescence microscopy (Axiovert 200 M and LSM 510 Meta, Zeiss). For quantification, the PKH26 fluorescence in cells and supernatant was measured by flow cytometry.

### Statistical analysis

Graphs were created with GraphPad Prism for Windows (version 5.04, GraphPad software). Data are means±SD and were compared with the two-tailed Student's t-test, p-values <0,05 were considered significant.

## Supplementary Figures and Tables




